# Small noncoding RNAs play superior roles in maintaining hematopoietic stem cell homeostasis

**DOI:** 10.1097/BS9.0000000000000123

**Published:** 2022-07-14

**Authors:** Hui Wang, Wenchang Qian, Yingli Han, Pengxu Qian

**Affiliations:** a Center of Stem Cell and Regenerative Medicine, and Bone Marrow Transplantation Center of the First Affiliated Hospital, Zhejiang University School of Medicine, Hangzhou, China; b Liangzhu Laboratory, Zhejiang University Medical Center, Hangzhou, China; c Institute of Hematology, Zhejiang University and Zhejiang Engineering Laboratory for Stem Cell and Immunotherapy, Hangzhou, China

**Keywords:** Hematopoietic stem cell, Homeostasis, Self-renewal, Small noncoding RNAs

## Abstract

The maintenance of the mammalian blood system depends on hematopoietic stem cells (HSCs), which are a rare class of adult stem cells with self-renewal and multilineage differentiation capacities. The homeostasis of hematopoietic stem cells is finely tuned by a variety of endogenous and exogenous regulatory factors, and disrupted balance will lead to hematological diseases including leukemia and anemia. Recently, emerging studies have illustrated the cellular and molecular mechanisms underlying the regulation of HSC homeostasis. Particularly, the rapid development of second-generation sequencing technologies has uncovered that many small noncoding RNAs (ncRNAs) are highly expressed in HSCs, including snoRNAs, miRNAs, tsRNAs, circular RNAs, etc. In this study, we will summarize the essential roles and regulatory mechanisms of these small ncRNAs in maintaining HSC homeostasis. Overall, this review provides up-to-date information in the regulation of HSC homeostasis by small ncRNAs, which sheds light into the development of therapeutic strategies against hematopoietic malignancies.

## 1. INTRODUCTION

Hematopoietic stem cells (HSCs) were first discovered in the early 1960s by 2 Canadian scientists—Ernest McCulloch and James Till. They discovered a small population of cells that could colonize the spleen while preserving the function of self-renewal and differentiation, and these cells are now called HSCs.^1^ Then, Ernest McCulloch and James Till discovered the potential clinical applications of HSCs in treating various hematological disorders and established the theoretical foundations for bone marrow transplantation, which thus far has benefited millions of patients with hematological malignancies by expanding their lifespan. These pioneering work of Ernest McCulloch and James Till was the foundations for the theory of HSC homeostasis, which holds that the characteristics of self-renewal and differentiation of HSCs are finely tuned and should be kept under precise balance, and any disturbance would lead to hematological diseases.

Over 1.3 million patients were diagnosed with hematological malignancies (including leukemia, lymphoma, and myeloma) worldwide in 2021,^2^ accounting for 7% of all newly diagnosed cancer patients. Hematopoietic malignancies are characterized by dysregulation of hematopoiesis, which could occur in the bone marrow, spleen, lymph nodes, and other tissues.^3–9^ If the self-renewal of HSCs goes awry or the differentiation is blocked, there will be too many preleukemic HSCs crowded in the bone marrow and no sufficient supply of mature blood cells, which is often the case of patients with leukemia. On the other hand, deficiency in HSC self-renewal would lead to exhaustion of HSC pools, resulting in anemia or cytopenia.^10^ Until now, regulation of HSC homeostasis is still the research focus, and the development of high-throughput sequencing technologies has greatly bolstered the studies of HSC homeostasis as well as underlying mechanisms. Since the complement of “Human Genome Project,” people realized that protein-coding genes only account for no >5% of all transcripts, the rest of which are all noncoding regulatory RNAs. Accumulating evidence implied the importance of noncoding RNAs in the regulation of HSC homeostasis, and in this study, we mainly summarize and discuss how small noncoding RNAs orchestrate HSC homeostasis.

## 2. SNORNAS IN THE REGULATION OF HSC HOMEOSTASIS

SnoRNAs are kinds of small RNA molecules that are relatively conserved and mainly exist in nucleoli, with length of about 50 to 250 nt. SnoRNAs can combine with other proteins, such as FBL and DKC1, to form snoRNP complex, which posttranscriptionally modifies rRNA or snRNAs. Classical snoRNAs are mainly divided into 2 types: C/D box (SNORD) and H/ACA box (SNORA). The former mainly mediates the 2′-O-me modification of target RNAs,^11^ while the latter mainly mediates the formation of pseudouridylation.^12^

At present, there are few reports on the regulation of hematopoietic stem cell homeostasis by snoRNAs. A few studies compared the differential expressions of snoRNA under physio/pathological conditions and found that the expression patterns of snoRNA under different pathological conditions exhibit specific characteristics. Warner et al^13^ compared the expression levels of snoRNA in 33 cases of acute myeloid leukemia (AML) and 6 normal blood samples, and found that a series of snoRNA molecules were abnormally expressed in AML patients, of which 37 snoRNAs were located in the DLK1-DIO3 or SNURF-SNRPN imprinted regions. Valleron et al^14^ found that the expressions of snoRNAs in SNORD112-114 cluster were significantly increased in patients with acute promyelocytic leukemia (APL), which was correlated with PML-RARα. These snoRNAs are all located in the DLK1-DIO3 region. However, other studies brought up different opinions on the mechanism of abnormal expression of SNORD112-114 cluster in APL patients. Cohen et al^15^ compared the expression levels of snoRNAs in peripheral blood cells of patients with APL, AML, and multiple myeloma. They found that the expression of SNORD113-114 cluster in the imprinted region DLK1-DIO3 was significantly increased. However, one patient did not carry PML-RARα. The authors speculated that the upregulation of snoRNA expression was not restricted to PML-RARα mutation, but RARα plays a crucial role.^15^ Additional research identified snoRNA expression signatures in other hematological malignancies, such as chronic lymphocytic leukemia (CLL) and pre-B-ALL.^16,17^ These studies identified characteristic snoRNA expression patterns in different types of leukemia, offering theoretical evidence for clinical application of snoRNAs in disease diagnosis and prognosis. Meanwhile, these studies demonstrated that snoRNAs play an important regulatory role in HSC homeostasis, as well as the occurrence and progression of different hematological malignancies.

SnoRNAs mainly function as guide to introduce 2′-O-me or pseudouridylation modification to target RNAs at specific sites, which are the most extensively distributed modifications in rRNAs.^18^ Fibrillarin (FBL) is the methyltransferase that catalyzes 2′-O-me. Besides, recent studies have found that FTSJ3 can also catalyze the 2′-O-me modification of rRNA,^19^ and the pseudouridine modification is catalyzed by DKC1 (dyskerin 1). rRNA modification has an important impact on target rRNA structure, processing, and function. In functional areas of ribosome, such as peptidyl-transferase center (PTC) and decoding sites, there are a large number of aggregated rRNA modifications, possibly to safeguard the progress of protein translation.^20^

At present, >200 sites on rRNA have been found to be modified, of which about 106 are modified by 2′-O-Me.^13^ However, recent studies have found that not all sites are completely modified, and modification levels are tissue-/physiological state-dependent. Krogh et al^21^ used the RiboMethSeq method to detect the level of 2′-O-Me modification in Hela cells and found that about two-thirds of the 106 modification sites were fully modified, while the rest were incompletely modified. At the same time, the study found that Hela cells and HCT116 cells showed significant differences in the modification levels of about 20% of the sites.^21^ Erales et al^22^ and colleagues used siRNA to knock down FBL in Hela cells and detected the level of 2′-O-Me modification. They found that the modification on certain rRNA sites was reduced by nearly 50%.^22^ Sharma et al^23^ also detected altered levels of modification at some rRNA sites in p53 knockdown cells.

These studies demonstrated that rRNA modification showed significant differences in different tissues and physio/pathological conditions. Meanwhile, these discoveries also indicate that there exists heterogeneity in cell ribosomes, that is, differential rRNA modification-resulted ribosome subpopulations with differentiated structures and functions. Ribosomal heterogeneity indicates that cells can tolerate the changes of rRNA modification to a certain extent, but the molecular mechanism and physiological significance are still elusive. Recent studies have found that ribosome heterogeneity is related to a variety of physiological and pathological processes. In some diseases, such as Diamond-Blackfan anemia (DBA), there are ribosomes with abnormal structure and function in patients, and such diseases have a high risk of cancer transformation.^24^ Ribosomal heterogeneity affects the translation of different mRNA, and rRNA modification is one of the sources of ribosomal heterogeneity. Therefore, it is reasonable that rRNA modification can also affect the level of ribosomal translation and participate in the regulation of a variety of physio/pathological processes, including HSC homeostasis. In Erales’ study, they found that change in the rRNA modification level led to change in some ribosomal functions, and specifically affected the translation initiation of ribosomes at internal ribosome entry site (IRES).^22^ Similarly, Zhou et al^25^ found that AML1-ETO mutation induced leukemia dependent on amino terminal enhancer of split (AES) protein, which enhanced the expression of many C/D box snoRNAs by binding to DDX21. In AML patients with AML1-ETO mutation, the expression of multiple snoRNAs increased significantly, and the modification level at corresponding rRNA sites also increased differentially. Among them, the modification levels of C1703 and G1328, which were related to Kasumi-1 cell self-renewal, increased most significantly, and the protein synthesis ability also increased accordingly.^25^

**Figure 1.**
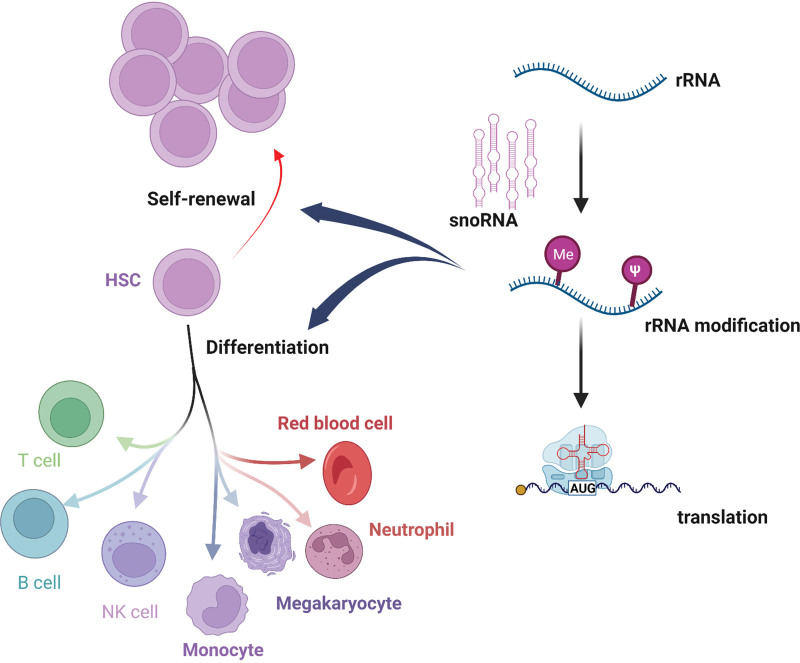
snoRNA in the regulation of HSC homeostasis. HSC = hematopoietic stem cell.

Based on the above studies, it is very likely that the rRNA modifications are tissue- and physiological state-dependent. Ribosomes in the cytoplasm are heterogeneous, and their substrate selection preferences affect the translation of different transcripts. Changes in the modification level of rRNA at different sites are one of the important sources of ribosomal heterogeneity. The expression level of snoRNAs can also affect the ribosome heterogeneity and regulate many physiological processes including HSC homeostasis through protein translational regulation (Fig. 1). Reported snoRNAs involved in hematopoietic malignancies were listed in the Table 1.

**Table 1.** SnoRNAs involved in hematopoietic malignancies.

SnoRNAs	Function	Ref.
snoRNA cluster in Dlk1-Dio3 region	Downregulation in AML	^13^
snoRNA cluster in SNURF-SNRPN region	Downregulation in AML	^13^
snoRNA cluster in Dlk1-Dio3 region	Upregulation in APL	^14,15^
scaRNA6, scaRNA9, SNORD49A, -55, -105	Upregulation in T-ALL compared with pre-B-ALL	^16^
SNORD24, SNORD44, SNORD82, SNORD110	Downregulation in T-ALL compared with pre-B-ALL	^16^
SNORA31, -6, -62, 70F, -71C	Downregulation in CLL	^17^
SNORA74A, SNORD116-18	Lower expression associated with shorter PFS	^17^
SNORD14D, 34, 35A, 43	Promoting translation and tumorigenesis of AML	^25^

ALL = acute lymphoblastic leukemia, AML = acute myeloid leukemia, CLL= chronic lymphocytic leukemia, PFS = progression free survival.

## 3. MIRNAS IN THE REGULATION OF HSC HOMEOSTASIS

MicroRNA (miRNA) is one of the noncoding RNAs of 19 to 22 nt in length. They target mRNAs by seed region to repress the translation or process cleaving of the target mRNAs, which regulate gene expression posttranscriptionally.^26^ The first miRNA was discovered in *Caenorhabditis elegans*, named Lin-4, in 1993. Till now, over 17,000 miRNAs have been found in >140 species, including humans.^27–29^ Each human miRNA is predicted to regulate thousands of mRNAs according to miRbase (https://mirbase.org/), and many of them are validated by several experimental methods, which imply us that miRNAs participate in diverse cellular and metabolic pathways in normal and malignant hematopoiesis, via the regulation of their downstream target genes.

It was reported that the miRNA expression in the hematopoietic system exhibited its unique pattern. A comprehensive analysis of the miRNA expression in the human hematopoietic system and its associated diseases indicated that 5 miRNAs, miR-142, miR-144, miR-150, miR-155, and miR-223 are predominantly expressed in hematopoietic cells.^30^ Several studies indicate unique miRNA expression patterns in different populations of hematopoietic hierarchy in mice or human. Sensitive and specific microfluidic qPCR was applied in mapping the miRNA expression in 27 hematopoietic subsets in murine. Seven miRNAs were identified highly expression in stem cell and progenitor populations. By contrast, 8 miRNAs expressed widely across differentiated cell types were at a low level in primitive cells.^31^ High expression of miR-125a-5p, miR-196b, miR-99a, miR-130a, and miR-125b-5p was identified by microarray in lineage^−^ cKit^+^ Sca1^+^ (LSK) cells from adult C57BL/6 mice. Most of the evolutionarily conserved miRNAs were both enriched in murine HSCs and human CD34^+^ HSPCs.^32^ Some exceptions, such as miR-142 and miR-181, were also reported. miR-142 expressed in human granulocytes and T cells was absent in monocytes and B cells, which was opposite in murine cells. MiR-181 was expressed in normal human B cells, T cells, monocytes, and granulocytes, but not in murine B cells by contrast.^33^

Specific miRNAs also play critical roles in different stages of hematopoiesis, including myeloid, lymphoid differentiation, and immune response. miR-9 accelerates terminal myelopoiesis through targeting Forkhead box class O genes (FoxOs), which was reported to inhibit myeloid differentiation.^34^ The recent study revealed the dynamic miRNAs expression in human erythroid cell differentiation using DNA Nanoball small RNA sequencing. MiR-126-3p was found to be the most abundant miRNA in early-stage samples with hCD34 and HUDEP-2 expression, while the most widely expressed miRNA in the late-stage samples was miR-451a.^35^ mir-150 regulates B-cell maturation by targeting c-MYB, which was reported to be related in lymphocyte development.^36,37^ miR-342 was recently reported to reduce pre-B-cell output by targeting lymphoid signaling pathways.^38^ HSCs with miR-125b over expression exhibited increased self-renewal and transplant ability.^39,40^ miR-125b was also related in the generation of activated nature of macrophages by the targeting IRF4.^41^ mir-223 was reported in regulating progenitor proliferation, granulocyte differentiation, and its activation.^42^

**Figure 2.**
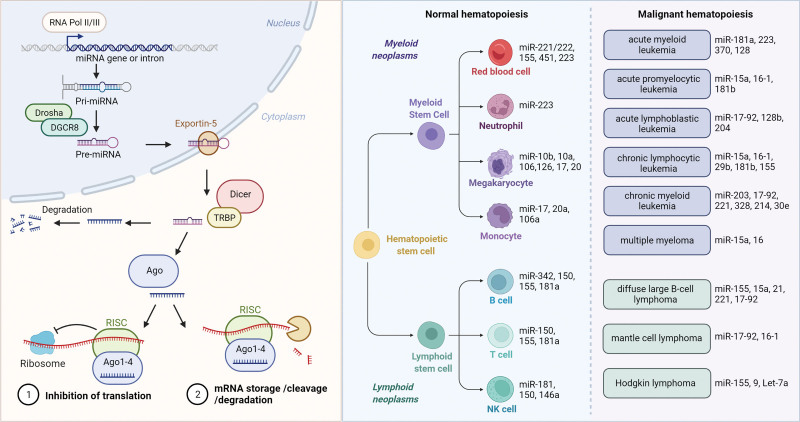
miRNAs in the regulation of HSC homeostasis. (Left) Pri-miRNAs are cleaved into a stem-loop structured miRNA precursor (pre-miRNA) by Drosha-DGCR8 complex and then exported into the cytoplasm. After cleavage by the Dicer/TRBP complex, the functional strand of the mature miRNA is loaded together with Argonaute (Ago2) proteins into the RNA-induced silencing complex (RISC), whereas the passenger strand is degraded. (Right) miRNAs in normal and malignant hematopoiesis. HSC = hematopoietic stem cell.

A plurality of causes and complicated mechanisms of malignant hematopoiesis have been extensively studied for a long time. The aberrant expression of miRNA was one of the reasons for the accumulation of distinct cell populations of the hematopoietic system, which are thought to be some of the reasons for leukemia and lymphoma. Numerous studies implied that miRNAs play key roles in the initiation and progression of different kinds of hematological malignancies. They were usually accompanied by abnormal expression of miRNAs so that some of these miRNAs are considered as diagnostic markers. Recently, the reduction of miR-32, miR-98, and miR-374 was found in the samples of CLL.^43^ Forty-four miRNAs were previously reported to generate a unique follicular lymphoma signature.^44^ miR-155 was found to be upregulated in preleukemic HSCs of AML patients carrying FLT3-ITD and NPM1 gene mutations.^45^ miR-223 inhibited cell proliferation and increased cell apoptosis in AML through FBXW7.^46^ MicroRNA-221 reduced the resistance to imatinib in chronic myeloid leukemia cells via targeting STAT5.^47^ These findings provide us a better understanding of the critical roles of miRNAs in leukemia, and the underlying mechanism to guide clinical application. Taken together, miRNAs play important roles in normal^33,36,37,42,48–60^ and malignant hematopoiesis,^61–74^ and the miRNA atlas will continue to be accomplished in utility as sequencing technique and research methods update (Fig. 2).

## 4. TSRNAS IN THE REGULATION OF HSC HOMEOSTASIS

Recent studies revealed mounting evidence regarding essential roles of tRNA-derived small RNA fragments, namely tsRNAs, in the regulation of hematopoiesis. tsRNAs are classified according to their relative locations to the parental tRNA isoacceptor sequence: 5′-tsRNA, 3′-tsRNA, and internally derived tsRNAs (i-tsRNA).^75,76^ Besides, tsRNAs are also categorized to constitutively expressed tsRNAs, most of which are similar to miRNAs in length, and stress-induced tsRNAs, which are usually longer (30–40 nt).^77,78^ It is worth clarifying that, according to previous studies, only ~1% of tRNAs are cleaved and processed into tsRNAs under stress conditions, meaning that tsRNAs production could not significantly affect tRNA pools inside the cells.^79^

Accumulating evidence unravel the crucial roles of tsRNAs in a multitude of biochemical processes, including translation,^78,79^ mRNA decay^80^ and location,^81^ and regulation of transposon activity.^82^ Moreover, expression of tsRNAs is dynamic and dysregulation of tsRNA expression is observed in various diseases,^83,84^ further implying its importance. The involvement of tsRNAs in regulation of hematopoiesis is gaining more and more attention. Guzzi et al reported that PUS7, the pseudouridine synthase of tRNA and mRNAs, was specifically upregulated in human HSCs, and its expression gradually decreased along with HSC differentiation, implying its involvement in regulation of HSC self-renewal. Specifically, depletion of PUS7 in HSCs led to decreased expression of tRNA-derived fragments, mTOG (mini 5′ terminal oligoguanine), as well as according pseudouridylation at specific sites. Interestingly, mTOG suppressed cap-dependent translation via binding to PABPC1 and thus disrupted translation initiation complex 4F. As a result, knockout of PUS7 resulted in increased cap-dependent translation, which is responsible for translation of many crucial transcripts sharing 5′ UTR with consensus characteristics. Moreover, depletion of PUS7 significantly impaired HSC self-renewal and differentiation, which could be rescued by ectopic expression of mTOG. PUS7 was also found downregulated in high-risk MDS patients, and ectopic expression of mTOG markedly potentiated self-renewal and corrected differentiation bias in HSPCs of MDS patients.^84^ Another study reported that knockout of DNMT2, responsible for m5C modification of tRNA-Asp, tRNA-Val, and tRNA-Gly, resulted in increased tsRNAs in the bone marrow of mice and, finally, led to HSC defects. Mechanistically, depletion of DNMT2 affected translation fidelity due to loss of tRNA-Asp methylation and impacted translation of specific subset mRNAs.^85^ Therefore, these works indicated that tsRNAs involve in the regulation of hematopoiesis in a modification-dependent manner.

It was reported that tsRNAs could also affect HSC homeostasis through modulating HSC microenvironments. A recent study elegantly showed that expression of angiogenin, responsible for cleavage of tRNA and production of tsRNA, was differentiated among subsets of mesenchymal stromal cells (MSCs), and secretion of angiogenin could modulate HSC functions.^86^ This was in accordance with subsequent work using angiogenin knockout mice, which observed fundamental expansion defects in LT-HSCs due to angiogenin deficiency in MSCs. Mechanistically, loss of angiogenin led to increased translation efficiency, which could be rescued by re-introducing according tsRNAs.^87^ In sum, these works highlighted the translation-suppressive roles of tsRNAs, especially for cap-dependent translation. This is in accordance with previous reports that most genes crucial for developmental processes, including hematopoiesis, share 5′ UTRs with common characteristics, and their translation mainly depend on cap-dependent translation.^88^ Taken together, roles of tsRNAs in the regulation of HSC hematopoiesis warrant further exploration.

## 5. CIRCULAR RNAS IN THE REGULATION OF HSC HOMEOSTASIS

Circular RNAs (circRNAs) are single-stranded, and closed-ring RNAs without poly-A tail, which were initially discovered in the 1970s.^89^ CircRNAs derive from transcripts that are connected through the backsplicing process. Circulation confers these RNAs with extra resistance to many kinds of RNase; thus, circRNAs are more stable than linear RNAs. As a result, although expressed in relatively low abundance, circRNAs are more persistent compared with their linear cognates.^90,91^ CircRNAs can form by multicombinations of exons and introns, so the length of circRNAs varies dramatically, depending on the splicing processes. Besides, the backsplicing process is also dynamically regulated, and multiple circRNAs could be produced from the same genes via alternative splicing.^92^

Similar to lncRNAs, emerging evidence demonstrated that circRNAs could actively take part in multiple posttranscriptional regulation of gene expression, via interaction with miRNAs, proteins, and other kinds of RNAs, and they could even generate small peptides via cap-independent translation.^93–95^ The roles of circRNAs in the regulation of hematopoiesis were also reported recently. Caldas et al^96^ first reported that splicing of MLL gene could result in complex products, some of which were circRNAs that may possess important functions during hematopoiesis. Then, in 2012, Salzman et al^91^ reported that in B-ALL patients, a bunch of circRNAs was specifically expressed, implying their potential as diagnostic biomarkers and/or therapeutic targets. Another comprehensive study compared circRNA expressions in HSPC, myeloid, lymphoid, and HEK293 cells. A total of 1950 circRNAs were detected, many of which were exclusively expressed in specific cell types, indicating that circRNAs are cell-type-specific and may involve in HSC homeostasis.^97^ Furthermore, it was reported that expression levels of circRNAs increased with progression of HSC differentiation into multiple lineages, and the amount of different types of circRNAs also increased dramatically, peaking in enucleated cells, platelets, and erythrocytes.^98,99^ And this also implied the importance of alternative splicing during HSC differentiation, since circRNAs are also generated from this process. Among them, circ-FIRRE and circ-BACH1 were specifically expressed in HSPCs, while circ-FUNDC3B, circ-MYBL1, and circ-AKT3 were preferentially expressed in mature blood cells.^98^

On the other hand, more explorations regarding function and mechanisms of circRNAs are needed in the future. Whether the specific expression signature contributes to HSC hematopoiesis is still unclear and warrants further study. The abnormal high abundance of circRNAs in platelet, and erythrocytes, and enucleated cells are also intriguing. Due to the relative high stability of circRNAs, they are considered as potential biomarkers in hematological malignancies as well as other diseases. Further functional studies may extend their potential for clinical applications.

## 6. CUTTING-EDGE TECHNOLOGIES FACILITATE THE STUDY OF SMALL NCRNAS IN HEMATOPOIESIS

Next-generation RNA sequencing (RNA-seq) technologies have been applied in diverse experimental and clinical studies for >10 years, which were considered as versatile and accurate methodologies to analyze changes of the whole transcriptomes among samples.^100,101^ The transcriptome sequencing techniques include mRNA-seq, small RNA sequencing (sRNA-seq), and long noncoding RNA sequencing (lncRNA-seq). mRNA-seq and lncRNA-seq provide the gene expression profiles, as well as the regulatory association between lncRNAs and targeted mRNAs. However, partly due to the biased ligation of sequencing linkers to both ends of RNAs, mRNA-seq had a poor performance in the accurate quantification of short RNA fragment within a sample.^102^ Small RNA-Seq (sRNA-Seq) is a technique for high-throughput quantification of sRNAs with 18 to 35 nucleotides with unprecedented sensitivity and dynamic range.^103^ It replenished the libraries of sRNAs, including miRNAs, rRNAs, tRNAs, and snoRNAs, which play pivotal roles in epigenomic regulation, for further studies in diverse biological processes and human diseases. For example, a recent study using deep sequencing provided a systematical profiling of five ncRNA classes including miRNA, snoRNA, small nuclear RNA (snRNA), small Cajal body-specific RNA (scaRNA), and tRNA fragments in mice tissues including bone marrow, and found that the Terc was specific to cells of hematopoietic origin.^104^

The second-generation sequencing methods do not map repeat regions precisely due to the short reads. However, Nanopore sequencing established in recent years, which belongs to the third-generation sequencing methods, is able to read through repeated regions with the long-read up to 2 Mb. So it has met the requirement in epigenetic studies in life and biomedical science. DNA and RNA molecules were detected when flowing through a nanopore, the nano-scale hole, by an applied electric filed.^105–107^ Therefore, nanopore technology was able to detect and analyze single-molecule amino acid, DNA, RNA, as well as long nucleotide sequences with modifications including methylation sites.^108^ The application of nanopore sequencing was applied in the detection and characterization of chromosomal translocations in AML recently.^109^

The cellular diversity within organisms or tumor has raised the concern for years while the sequencing techniques are not designed to resolve mixed populations of cells. Single-cell sequencing (scRNA-seq) can be used to identify gene expression within a single population.^110,111^ Therefore, it allows us to explore the cell heterogeneity in the bulk population and trace cell fate to reconstruct developmental trajectories. Moreover, since human hematopoietic cells are difficult to obtain, the single-cell technique overcomes the problem of insufficient sample sizes, allowing studies previously performed in mouse models to be recapitulated in human cells. scRNA-seq performed outstanding in detecting complicated factors leading to rare but critical subpopulations that determines KMT2A-r ALL patient outcome.^112^ The essential cross-talk between CAR-T subsets and the tumor microenvironment was well illustrated in an immunocompetent B-cell lymphoma mouse treated with anti-CD19 CAR-T therapy.^113^ Single-cell technologies were also employed in constructing AML hierarchies, including cell fates, regulation factors, and specific gene expression with implications for precision medicine and immune therapies.^114^ Besides, single-cell assay has been modified to detect epigenetic status, such as chromatin accessibility, histone modification, and DNA methylation. Integrative scRNA-seq combined with the single-cell transposase-accessible chromatin sequencing (scATAC-seq) was applied in the analysis of human developmental hematopoiesis. The chromatin accessibility and the specific transcription factors presented almost opposite in HSPCs compared with the mature lineages.^115^ Single-cell nucleosome, methylation, and transcription sequencing (scNMT-seq) which labels open chromatin by a methyltransferase were applied in mESCs to achieve the profiling of DNA methylation and transcription in a small number of stem cells during embryonic differentiation.^116^ Single-cell DNA methylome sequencing was performed in human preimplantation embryos and de novo DNA methylation could be traced in early blastomeres.^117^ The aforementioned single-cell transcriptome and epigenome techniques may provide us with new tools for studying the roles of small ncRNAs in the regulation of HSC homeostasis.

CRISPR/Cas9-based gene screening, a large-scale unbiased genetic loss-of-function filtration method designed to unbiased screen functional genes, was also applied in investigating the epigenetic regulation in hematopoiesis and leukemia. With the various modifications on dCas9/sgRNA complex, CRISPR interference (CRISPRi), CRISPR activation (CRISPRa), and CRISPRon/off techniques were developed successively, while the first 2 were widely used for screening of noncoding RNAs.^118–120^ The CRISPR screening of epigenetic regulator revealed that MPP8 promoted AML progression. MMP8 inhibition led to the DNA damage and cell cycle exit in AML cells.^121^ METTL3 was identified as an essential gene via whole-genome CRISPR screen in AML cell growth which induced m6A modification in the coding region of mRNAs and enhanced its translation.^122^ A recent study demonstrated a robust platform for large-scale pooled CRISPRa along with CRISPRi to identify gene networks in primary human T cells.^123^ Creative researchers have combined some aforementioned techniques to solve epigenetic problems. CRISPRi or CRISPR knockout was combined with ATAC-seq in single cells, which named Perturb-ATAC in a recent study, to identify epigenomic functions of chromatin regulators and transcription factors, as well as noncoding RNAs in B cells.^124^

In summary, the application of cutting-edge techniques could assist us to further appreciate the roles and epigenetic mechanisms of small noncoding RNAs in hematopoietic homeostasis and diseases. Innovation in the development of methods will offer us more opportunities and tools to resolve the long-standing puzzles in the future.

## 7. CONCLUSION AND FUTURE PERSPECTIVES

In this study, we have summarized recent literature and discussed the essential roles and regulatory mechanisms of small ncRNAs, including snoRNAs, miRNAs, tsRNAs, and circRNAs, in maintaining HSC homeostasis. Recently, advances in single-cell sequencing technologies have greatly deepened our understanding of HSC homeostasis. Nevertheless, applications of scRNA sequencing in ncRNAs and RNA modifications are often limited due to technical challenges. For example, miCLIP and RiboMethSeq are the techniques used to detect m6A and 2′-O-Me, respectively, but neither of them could be performed at the single-cell level. Recently, Yin et al^125^ reported SLIM-seq method, which can detect m6A marks in the whole transcriptome using very few HSPC cells. Using this technique, they profiled dynamic landscape of m6A modification during HSPC differentiation, which further deepened our knowledge of m6A modification in the HSC homeostasis.^125^ Besides, Ai et al^126^ reported an itChIP-seq method, which can detect chromatin modification states at the single-cell level. In the future, single-cell small noncoding RNA sequencing technologies with higher resolution and lower input demand would greatly deepen our understanding of the roles of small ncRNAs in HSC homeostasis. In summary, this review provides up-to-date information on the regulation of HSC homeostasis by small ncRNAs, which sheds light into the development of therapeutic strategies against hematopoietic malignancies.
